# Preventing Antiretroviral Treatment Interruptions among HIV/AIDS Patients in Africa

**DOI:** 10.1371/journal.pmed.1001370

**Published:** 2013-01-08

**Authors:** Edward J. Mills, Christine Nabiryo

**Affiliations:** 1 Faculty of Health Sciences, University of Ottawa, Ottawa, Canada; 2 Stanford Prevention Research Center, Department of Medicine, Stanford University School of Humanities and Sciences, Stanford, California, United States of America; 3 The AIDS Support Organization (TASO), Kampala, Uganda

## Abstract

Edward Mills and Christine Nabiryo discuss a new qualitative study in *PLOS Medicine* that offers insight into disengagement from HIV treatment care in sub-Saharan Africa.

**Linked Research Article**

This Perspective discusses the following new study published in *PLOS Medicine*:

Ware NC, Wyatt MA, Geng EH, Kaaya SF, Agbaji OO, et al. (2013) Toward an Understanding of Disengagement from HIV Treatment and Care in Sub-Saharan Africa: A Qualitative Study. PLoS Med 10(1): e1001369. doi:10.1371/journal.pmed.10001369

Norma Ware and colleagues conducted a large qualitative study among patients in HIV treatment programs in sub-Saharan Africa to investigate reasons for missed visits and provide an explanation for disengagement from care.

The roll-out of antiretroviral therapy (ART) in the poorest parts of the world is one of the greatest public health achievements in history. During the 1990s in Africa and elsewhere, thousands died every day for lack of access to treatment. Back then, courageous flight attendants at Lufthansa airlines, couriers at DHL company, and family members from the African diaspora found clever, albeit not always legal, ways to smuggle antiretrovirals into Africa and keep small numbers of patients alive. Visionary thinkers in organizations such as Médecins Sans Frontières (MSF) in South Africa and the Joint Clinical Research Centre (JCRC) in Uganda began treating patients while donors prevaricated over concerns about drug resistance, adherence challenges, and human resources logistics [1]. Then, in 2003, thanks in large part to US President George W. Bush, the President's Emergency Plan for AIDS Relief (PEPFAR) and The Global Fund began providing widespread antiretrovirals to those qualifying as most in need of treatment [2]. This signaled a massive turning point in the pandemic. The challenge now would be getting people onto treatment and keeping them on treatment. A new study in this week's *PLOS Medicine* [3] offers more insight into this challenge.

Historically, one issue that was not foreseen was that despite the best efforts of providers and donors, a proportion of patients would withdraw from treatment. Sydney Rosen and colleagues published their important systematic review on the prevalence of loss to follow up among early treatment cohorts in 2007 [4]. Their study reported average attrition from early treatment programs at about 40%, and spirited the development of a field of research examining rates and risk factors for retention in programs. This was a wake-up call to those working on issues of adherence that pillboxes and fixed-dose combinations do nothing to help a patient who is no longer in care.

Even now, our understanding of why people drop out of care, and how to intervene, remains limited [5]. Initially, there was widespread concern that most patients who leave care have died. However, studies that tracked patients lost from care have since provided important insights [6], including the fact that mortality among patients lost to follow up in Africa varies widely. Early tracing studies, for example, found that 12%–87% of patients died, indicating large heterogeneity [6]. Mortality associated with attrition from care is inversely associated with the proportion of patients lost [7]. So, if patients have disengaged from care and are not dead, then why have they chosen to interrupt life-saving treatment?

Structured supervised treatment interruptions were once explored as a way to ration therapy and minimize adverse events but were found to result in higher mortality, and this strategy was abandoned [8]. A systematic review found that about 23% of patients take an unstructured treatment interruption that lasts an average of 150 days [9]. Interruptions from clinical care can be dichotomized between short-term and long-term. Short-term interruptions can be characterized as patients who miss clinical appointments due to a logistic or health barrier and will re-enter care when they can. These patients will likely miss both clinical assessment and take a short-term break from their pill-taking, increasing the risk of developing drug resistance, but ultimately returning to care. Longer-term interruptions may have more grave consequences. In an assessment of longer-term retention in care in Cameroon [10], Mégane Meresse and colleagues found that, among patients on treatment in the longer term, 20% had experienced treatment interruptions and these were uniquely predictive of virological failure, whereas adherence assessments did not predict failure.

For many reasons, the study in this week's *PLOS Medicine* by Norma Ware and colleagues will be important for those engaged in supporting patient care in resource-limited settings. This study helps us understand that patient interactions with clinics are not simply about receiving pills or seeing a doctor. Some patients interrupt care due to costs or logistical reasons and likely re-enter care when their situation improves, while others discontinue care because of a lack of connection with their fellow patients or caregivers. These are different problems requiring different solutions. Even when field officers traced patients in this study, some patients felt that they were viewed as being bad patients. This should be a learning opportunity for those of us working in the HIV/AIDS field. An HIV+ diagnosis is often associated with a sense of shame and judgment that the person has engaged in some act that is socially unacceptable, whether that person has a partner who is unfaithful or themselves engage in casual sex. Ware and colleagues importantly highlight that, for patients who feel disconnected from a sense of community with the clinic, the shame and judgment are compounded at the clinic level and result in discontinuation of care.

Several organizations have endeavored to create a sense of community at the clinic level. Efforts to build a sense of connection include drama and music groups, income-generating activities, and community-based delivery of care [10]. While these may sound like unscientific methods to provide medical care, they recognize that patients who feel disconnected from a community will ultimately have poor clinical outcomes that affect them, their families, and the local community. One of the most important evaluations of a community-based strategy comes from a rural MSF program in Mozambique [11]. Tom Decroo and colleagues referred patients to self-forming groups of patients in rural communities whose responsibility was to look after each other through assessing each other's health, ensuring adherence, and retaining each other in care. After more than 1 year, among 1,384 patients initiated on ART, 2% had died, and only two individual patients (0.2%) were absent from care, compared with 20% in other local programs. These remarkable findings display the importance of patients caring for other patients through shared experiences, lessons about coping, and friendship. Could “expert patients” be the next specialized health worker in the pandemic? [12]

ART roll-out has been a highly innovative endeavor, and there have been many lessons learned since 2003, but there is still a long way to go. The next challenge is to find the best models for delivering effective ART care over the long-term. Understanding the reasons is a necessary first step, but this work should now move to interventional studies to define the most appropriate service delivery and patient support models for the next decade and beyond.

## Abbreviations

ART: antiretroviral therapy
MSF: Médecins Sans Frontières
